# Modeling of Thermal Phase Noise in a Solid Core Photonic Crystal Fiber-Optic Gyroscope

**DOI:** 10.3390/s17112456

**Published:** 2017-10-26

**Authors:** Ningfang Song, Kun Ma, Jing Jin, Fei Teng, Wei Cai

**Affiliations:** Department of Opto-Electronics Engineering, Beihang University, Beijing 100191, China; songnf@buaa.edu.cn (N.S.); kunma@buaa.edu.cn (K.M.); tengfei0337@126.com (F.T.); sdfz174caiwei@126.com (W.C.)

**Keywords:** thermal phase noise, fiber-optic gyroscope, solid core photonic crystal fiber

## Abstract

A theoretical model of the thermal phase noise in a square-wave modulated solid core photonic crystal fiber-optic gyroscope has been established, and then verified by measurements. The results demonstrate a good agreement between theory and experiment. The contribution of the thermal phase noise to the random walk coefficient of the gyroscope is derived. A fiber coil with 2.8 km length is used in the experimental solid core photonic crystal fiber-optic gyroscope, showing a random walk coefficient of 9.25 × 10^−5^ $\deg/\sqrt{h}$.

## 1. Introduction

Interferometric fiber-optic gyroscopes (IFOGs) measure the angular rate around a fixed axis based on the Sagnac effect, and have been increasingly used due to their particular properties such as high performance, low power consumption and high reliability as a solid state structure with no moving parts [1]. The typical implementation example is the LN-200 in ‘Clementine’ in 1994, which was the first IFOG utilization in aerospace navigation. With the development of inertial guidance and navigation systems, the accuracy of IFOGs is improving by using different signal processing and noise suppression methods, such as square-wave modulation, relative intensity noise (RIN) subtraction and modulation phase optimization [2,3]. According to a recent report, the MARINS series IFOGs of iXBlue.co have even reached a bias stability of 2 × 10^−5^ degrees per hour [4]. When the gyros are designed for high-sensitivity applications, the thermal phase noise seems to be a fundamental limitation. The thermal phase noise is usually caused by the thermal fluctuations in the refractive index of the fiber [5,6] and was first observed in a conventional fiber-optic gyro in [7]. Additionally, for an open-loop dynamically sinusoidal biased IFOG, it was demonstrated theoretically [8] and then experimentally verified by Moeller and Burns in 1996 [9]. However, in the abovementioned studies, they adopted single mode fibers (SMFs) and polarization maintaining fibers (PMFs), respectively, and there have been no prior studies regarding the thermal phase noise in solid core photonic crystal fiber-optic gyroscopes (SC-PCFOGs). In fact, during long-term exposure in a space environment, the components of the space used IFOG are generally affected by radiation, leading to the performance degradation of the system. Compared with the conventional PMFs, solid core photonic crystal fibers (SC-PCFs) has much lower sensitivity to temperature and radiation in the same conditions [10,11], which shows great prospect in space applications and SC-PCFOGs. As the SC-PCFs are different from conventional PMFs in structure and material, and the thermal phase noise can be the limitation of high-precision SC-PCFOGs, it is necessary to analyze and measure the thermal phase noise in SC-PCFOGs. 

In this paper, a theoretical model of thermal phase noise in a square-wave modulated SC-PCFOG was derived, and a thermal phase noise measurement setup was built to verify the model. During the measurement, a RIN subtraction method was adopted and the noise in the output signal decreased by 18 dB with this method. Additionally, the measured thermal phase noise voltage coincided with the theoretical thermal phase noise calculated from the derived model. Furthermore, contribution from thermal phase noise to the random walk coefficient of the gyroscope was calculated and showed a value of $9.25\times 10^{-5}\deg/\sqrt{h}$ using our SC-PCFOG parameters.

## 2. Theory of Thermal Phase Noise

**Figure 1:**
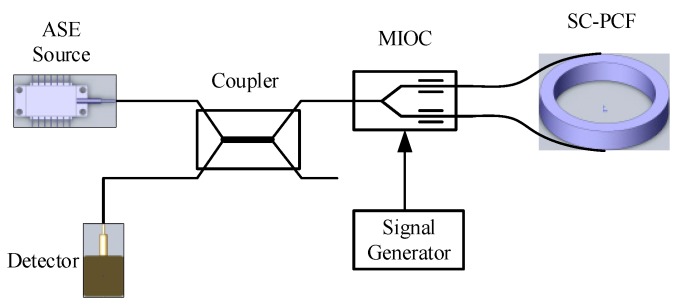
Schematic diagram of a solid core photonic crystal fiber-optic gyroscope.

As illustrated in Figure 1, a SC-PCFOG operating in a typical reciprocal configuration consists of an amplified spontaneous emission (ASE) source with high power and broad spectrum, a coupler, a multifunction integrated optical circuit device (MIOC) and a SC-PCF coil.

For a square-wave phase modulation applied on the MIOC, the interference output of the IFOG becomes [1]:

(1) $$P_{d}(t)=\frac{1}{2}P_{0}\{1+\cos[\Delta \phi_{R}(t)+\Delta \phi_{N}(t)+\Delta \phi_{B}(t)]\}$$

where $1/2P_{0}$ is the input optical power, *P_d_* is the optical power at the detector, $\Delta \phi_{R}$ is the rotation-induced phase difference, $\Delta \phi_{B}$ is the square-wave modulation bias phase and $\Delta \phi_{N}$ is the phase perturbation owing to noise. If we further assume that the gyro remains at rest, $\Delta \phi_{R}\approx 0^{{}^{\circ}},$ Equation (1) can be expressed as:

(2) $$P_{d}(t)\approx \frac{1}{2}P_{0}\left[1+\cos\Delta \phi_{B}(t)-\Delta \phi_{N}(t)\sin\Delta \phi_{B}(t)\right]$$

The MIOC is driven by an oscillator to generate a square-wave modulation $\Delta \phi_{B}=\pm \phi_{0}$, where $\phi_{0}$ is the amplitude of the square-wave. We can expand $\Delta \phi_{B}$ by Fourier series at a frequency $\omega_{m}=2\pi f_{m}$, $f_{m}$ is the modulation frequency:

(3) $$\begin{array}{l} \Delta \phi_{B}(t)=\sum_{n=1}^{\infty }\left[\frac{4\phi_{0}}{n\pi }\sin^{2}(\frac{n\pi }{2})\sin(n\omega_{m}t)\right]\\ =\frac{4\phi_{0}}{\pi }\sin(\omega_{m}t)+\frac{4\phi_{0}}{3\pi }\sin(3\omega_{m}t)+\ldots +\frac{4\phi_{0}}{(2n-1)\pi }\sin\left[(2n-1)\omega_{m}t\right],\;n=1,2,3\ldots .. \end{array}$$

Only the first three orders of Equation (3) were retained and substituted into Equation (2). Then through *J_n_* Bessel function expansion, we obtain:

(4) $$\begin{array}{cc} I_{d}(t)=\frac{1}{2}I_{0}\langle 1+ & \left[J_{0}(A)+2\sum_{n=1}^{\infty }J_{2n}(A)\cos(2n\omega_{m}t)\right]\times \left[J_{0}(\frac{A}{3})+2\sum_{n=1}^{\infty }J_{2n}(\frac{A}{3})\cos(6n\omega_{m}t)\right]\\ - & \left[2\sum_{n=0}^{\infty }J_{2n+1}(A)\sin\{\left(2n+1\right)\omega_{m}t\}\right]\times \left[2\sum_{n=0}^{\infty }J_{2n+1}(\frac{A}{3})\sin\{\left(6n+3\right)\omega_{m}t\}\right]\\ -\Delta \phi_{N}(t)\times & [2\sum_{n=0}^{\infty }J_{2n+1}(A)\sin\left\{(2n+1)\omega_{m}t\right\}\times \left\{J_{0}(\frac{A}{3})+2\sum_{n=1}^{\infty }J_{2n}(\frac{A}{3})\cos(6n\omega_{m}t)\right\}+\\ & \left\{J_{0}(A)+2\sum_{n=1}^{\infty }J_{2n}(A)\cos(2n\omega_{m}t)\right\}\times 2\sum_{n=0}^{\infty }J_{2n+1}(\frac{A}{3})\sin\left\{(6n+3)\omega_{m}t\right\}]\rangle , \end{array}$$

where the modulation depth $A=4\phi_{0}/\pi$. *I*_0_ and *I_d_* are the input and output optical intensities and can be expressed as *I = ηP*, where *η* is the detector responsivity. By performing a Fourier transform to Equation (4), and only considering the noise terms, the noise spectrum is given by:

(5) $$\left|I_{N}(\omega )\right|=\frac{1}{2}I_{0}\sqrt{4\pi BS}$$

(6) $$\begin{array}{cc} S & =J_{0}^{2}(A/3)\sum_{n=0}^{\infty }J_{2n+1}^{2}(A)\left\{\Delta \phi_{N,rms}^{2}\left[\omega +\left(2n+1\right)\omega_{m}\right]+\Delta \phi_{N,rms}^{2}\left[\omega -\left(2n+1\right)\omega_{m}\right]\right\}\\ & +J_{0}^{2}(A)\sum_{n=0}^{\infty }J_{2n+1}^{2}(A/3)\left\{\Delta \phi_{N,rms}^{2}\left[\omega +\left(6n+3\right)\omega_{m}\right]+\Delta \phi_{N,rms}^{2}\left[\omega -\left(6n+3\right)\omega_{m}\right]\right\}\\ & +\sum_{m=1}^{\infty }\sum_{n=1}^{\infty }J_{2m-1}^{2}(A)J_{2n}^{2}(A/3)\sum_{i=1}^{2}\sum_{j=1}^{2}\left\{\Delta \phi_{N,rms}^{2}\left[\omega +{\left(-1\right)}^{i}\left(2m-1\right)\omega_{m}+{\left(-1\right)}^{j}\left(6n\right)\omega_{m}\right]\right\}\\ & +\sum_{m=1}^{\infty }\sum_{n=1}^{\infty }J_{2m}^{2}(A)J_{2n-1}^{2}(A/3)\sum_{i=1}^{2}\sum_{j=1}^{2}\left\{\Delta \phi_{N,rms}^{2}\left[\omega +{\left(-1\right)}^{i}\left(2m\right)\omega_{m}+{\left(-1\right)}^{j}\left(6n-3\right)\omega_{m}\right]\right\} \end{array}$$

(7) $$\begin{array}{cc} \langle \Delta \phi_{N,rms}^{2}(\omega )\rangle & \approx \frac{k_{B}T^{2}L}{\kappa \lambda^{2}}{\left(\frac{dn_{eff}}{dT}+n_{eff}\alpha_{L}\right)}^{2}\times \\ & \ln\left\{\frac{{\left[{\left(\frac{2}{W_{0}}\right)}^{2}+{\left(\frac{\omega }{v}\right)}^{2}\right]}^{2}+{\left(\frac{\omega }{D_{i}}\right)}^{2}}{{\left[{\left(\frac{4.81}{d}\right)}^{2}+{\left(\frac{\omega }{v}\right)}^{2}\right]}^{2}+{\left(\frac{\omega }{D_{i}}\right)}^{2}}\right\}\times \left[1-\sin c(\frac{\omega L}{v})\right], \end{array}$$

Equation (5) represents the single-sided thermal phase noise in the IFOG when a square-wave bias signal is applied, where *B* is the electrical bandwidth. Equation (7) is phase noise spectral density caused by temperature induced index fluctuations in the fiber of a Sagnac interferometer as [7], where the length of fiber coil is defined by *L*, *ν* is the effective speed of light in the fiber, *λ* is the operational wavelength, *W*_0_ is the mode field radius and *d* is the cladding diameter of fiber. *α_L_* is the linear thermal expansion coefficient, *D_i_* is the thermal diffusivity, *κ* is the thermal conductivity, *k_B_* is the Boltzmann constant, *T* is the temperature and *dn_eff_/dT* is the temperature coefficient of the fiber effective refractive index in the fiber. By multiplying Equation (5) by the detector responsivity *η* and relating *I*_0_ to the optical power *P*_0_, we can get the thermal phase noise at the detector as:

(8) $$i_{N}(\omega )=\frac{1}{2}\eta P_{0}\sqrt{4\pi BS}$$

where *P*_0_ is the output optical power without modulation. For small rotation rates, the signal current *i_s_* is given by the coefficient of the first harmonic terms of Equation (4), as in the usual noise calculation in the appendix of [12]:

(9) $$i_{S}=\frac{\Delta \phi_{R}J_{1}\left(A\right)J_{0}\left(A/3\right)I_{0}}{\sqrt{2}}$$

By setting the signal current at the fundamental frequency component *ω_m_* equal to the thermal phase noise current and introducing the Sagnac phase shift, we can obtain the frequency-dependent minimum detectable rotation rate as:

(10) $$\Omega_{rms,thermal}(\omega )=\frac{\lambda c}{2\pi LD}\frac{1}{J_{1}\left(A\right)J_{0}\left(A/3\right)}\sqrt{2\pi BS}$$

where *D* is the diameter of fiber coil and *c* is the speed of light.

## 3. Experimental Setup

**Figure 2:**
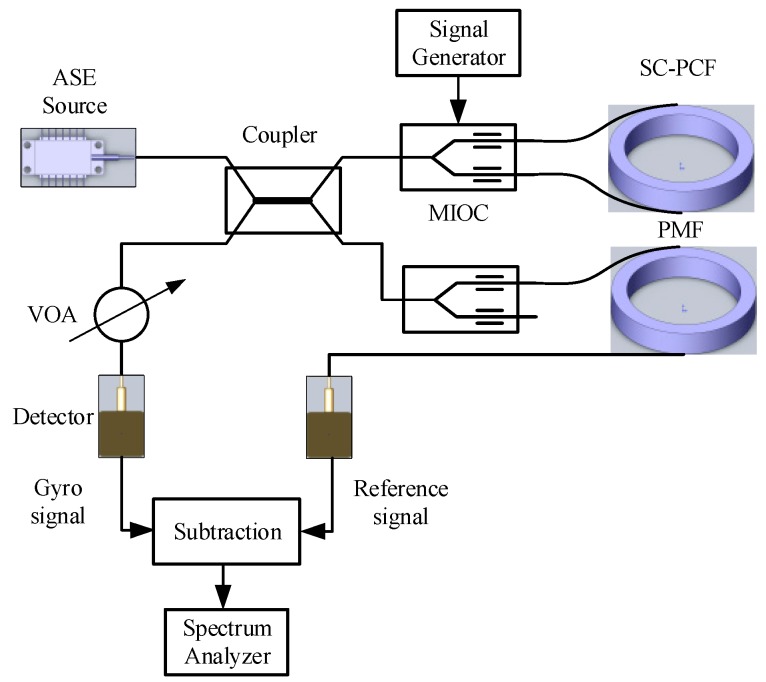
Experimental set-up for the measurement of the thermal phase noise in the SC-PCFOG.

The experimental set-up for measurement of thermal phase noise in the SC-PCFOG is shown schematically in Figure 2. The corresponding RIN subtraction method was adopted to produce a gyro signal with reduced RIN. Light from an ASE with a maximum output power of 4.8 mW was focused into the end of the input coupler, which delivered 5.85 μW at the gyro detector when we took into account the splice and optical components insertion losses. A MIOC was added on the unused lead of the 50/50 coupler in the reference arm to ensure that its transmission axis was parallel to the transmission of gyro arm. For maximizing the signal level, the square wave modulation phase was 1.37 rad at the proper frequency of the fiber coil. And the MIOC was operated in a push-pull mode, which led to a net phase bias of 7π/8. As the principle of RIN subtraction is to extract the reference signal from the unused lead of the couple, and to subtract it from gyro signal after latency through fiber, the reference signal was then delayed through a PMF coil equivalent to the length of the SC-PCF coil. An adjustable attenuator was then spliced to the output lead of the coupler. Both signals were detected by photo-detectors and the output signals were then amplified separately and subtracted in a differential amplifier. The output noise was measured by a spectrum analyzer.

**Figure 3:**
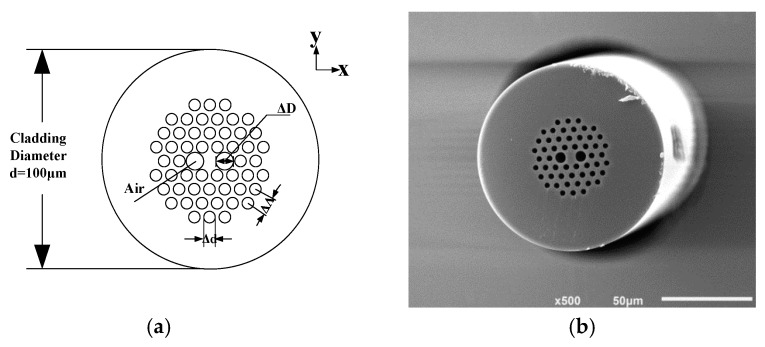
Solid core photonic crystal fiber used in the experiment (**a**) Drawing of the cross section (**b**) Scanning electron micrograph

All the parameters of experimental IFOG are listed in Table 1. As some fiber-optic parameters vary with fiber composition and geometry, the measured fiber structure parameters are also given in Table 1. The SC-PCF we used was fabricated in collaboration with Fiber Home Telecommunication Technologies CO., Ltd. The fiber cross section and the scanning electron microscope (SEM) photograph of the PCF are shown in Figure 3. Considering the applicability to the SC-PCF, we adopted the commercial software COMSOL Multiphysics 4.4 based on full vector finite element method to generate the numerical simulation of thermal conductivity *κ*, temperature coefficient of refractive index $dn_{eff}/dT$ and linear thermal expansion coefficient $\alpha_{L}$. The emulation values as well as typical parameters used in Equations (5)–(7) are presented in Table 1.

**Figure 4:**
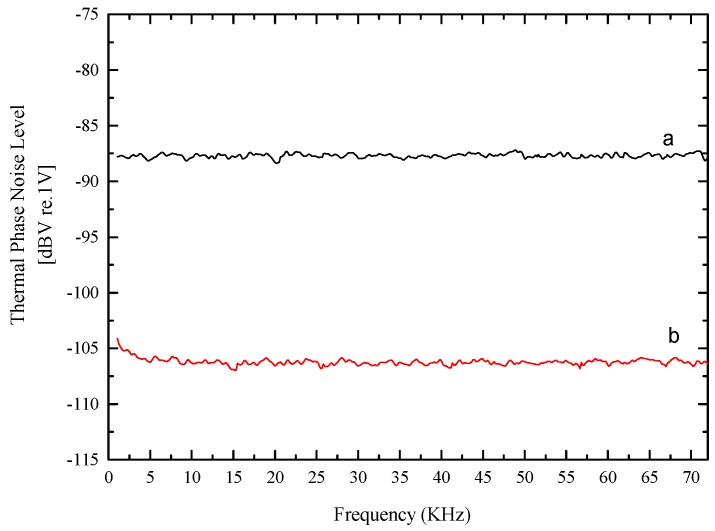
The output signals without phase modulation from spectrum analyzer: without noise subtraction (curve a), with noise subtraction (curve b).

The key of RIN subtraction is to ensure that the RIN of the reference signal and the gyro signal are coherent. Hence, prior to subtraction, the optical path difference (OPD) between gyro signal and reference signal was adjusted first to ensure the efficiency of RIN subtraction. The delay time between both signals can be obtained by estimating the position of the maximum correlation coefficient of the correlation array. In our experiment, when a 295.28 m of typical PM fiber was added to the reference arm, the OPD between gyro signal and reference signal was less than 8.28 cm. During the measurements, the optical power arriving at the detector was held constant by adjusting the attenuator, so that the reference signal and gyro signal had the same intensity but different optical paths. In Figure 4 the output signals are presented with and without noise subtraction respectively. Experimentally, we measured 18 dB reduction by subtracting the two outputs when the SC-PCFOG was without modulation, which quantified that the RIN subtraction scheme is available for our measurement.

## 4. Results and Discussion

**Figure 5:**
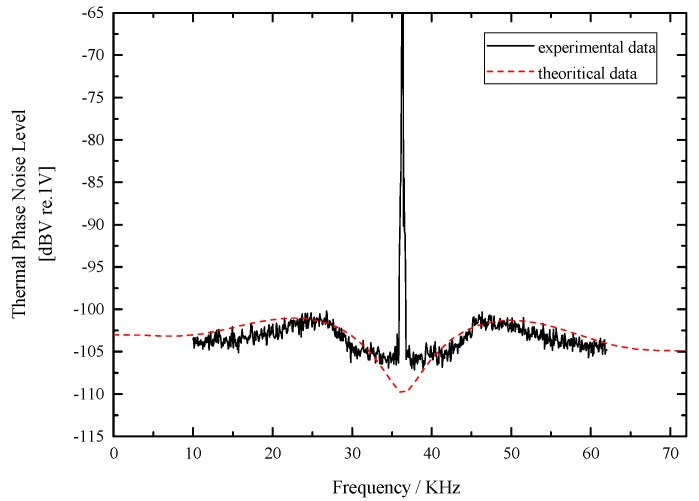
Experimental thermal phase noise voltages obtained with the spectrum analyzer with 1.37 rad square-wave modulation and the corresponding calculated result.

The measured noise and the theoretical thermal phase noise calculated from our model are presented in Figure 5. Due to the Earth’s rotation, the measured signal contains a peak at the modulation frequency (36.337 kHz). Despite this, the agreement between the calculated and experimental results is quite good with only 1–2 dB difference. This difference could be induced by the error of parameters and the experimental uncertainties.

**Figure 6:**
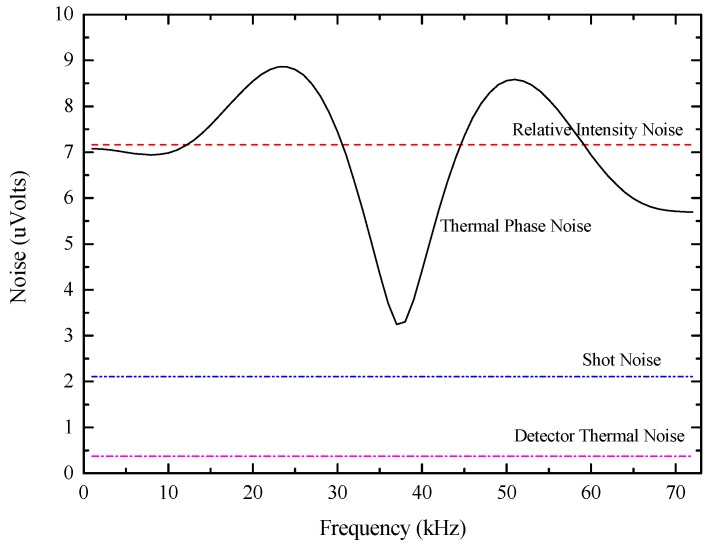
Calculated noise voltages for the solid core photonic crystal fiber-optic gyroscope.

The thermal phase noise, shot noise, RIN and detection thermal noise are calculated and then plotted in Figure 6. And the theoretical calculation formulas of above mentioned noises can be expressed as [13,14]:

(11) $$\sigma_{shot}^{2}=2eI_{p}B$$

(12) $$\sigma_{RIN}^{2}=I_{p}^{2}B/\Delta v$$

(13) $$\sigma_{T}^{2}=4kTB/R$$

where $\sigma_{shot}^{2}$, $\sigma_{RIN}^{2}$ and $\sigma_{T}^{2}$ represent shot noise power, RIN power and detection thermal noise power, respectively. Both the relative intensity noise and shot noise are calculated with the measured detector average current $I_{p}$ of 5.56 μA, while the bandwidth of the optical source Δv is 12 nm. And other parameters for the calculation are listed in Table 1.

**Figure 7:**
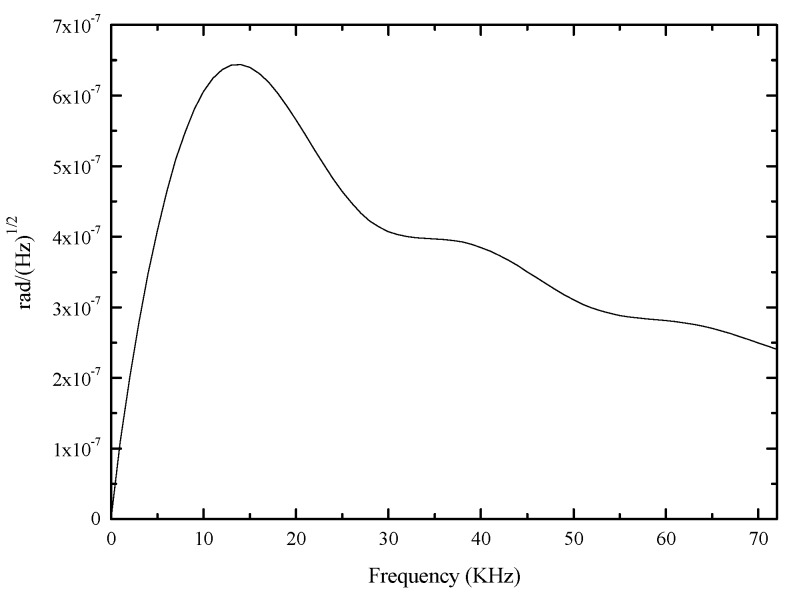
The phase noise spectral density as a function of frequency.

From Figure 6 we see that, for our configuration, the level of thermal phase noise is higher than shot noise and detection thermal noise, but lower than RIN at the modulation frequency. Clearly, just off the modulation frequency the wings of thermal phase noise are quite large. Based on the theoretical thermal phase noise model of Equation (7), the relationship of phase noise spectral density and frequency is represented in Figure 7. The phase noise spectral density at 1 Hz is 2.8 × 10^−9^ $\mathrm{rad}/\sqrt{\mathrm{Hz}}$, and the contribution from thermal phase noise in SC-PCFOG to random walk coefficient (RWC) can be obtained by $\Omega_{rms}/\sqrt{B}.$ According to Equation (10), the calculated result is 9.25 × 10^−5^ $\deg/\sqrt{h}$ which indicates the detection limitation of SC-PCFOG. 

## 5. Conclusions

A theoretical thermal phase noise model in a square-wave modulated SC-PCFOG was derived. The thermal phase noise in an experimental SC-PCFOG was measured, showing a good agreement between theory and experiment. The contribution of thermal phase noise to the RWC was also calculated, giving an estimate of the detection limit of SC-PCFOG as 9.25 × 10^−5^ $\deg/\sqrt{h}$. Compared with the RIN, the thermal phase noise is not the dominant noise source in SC-PCFOG, but it is of the same order of magnitude as the shot noise. For our experimental SC-PCFOG, RIN subtraction was necessary to measure the thermal phase noise and the results confirm that the thermal phase noise can be a fundamental limitation in the high-sensitivity SC-PCFOG when a RIN subtraction method is employed.

## Figures and Tables

**Table 1:** Parameters of the experimental IFOG used in the computations.

Parameter	Property	Value
*η* (A/W)	Detector responsivity	0.95
*R* (kΩ)	Load resistance	288
*λ* (nm)	Operational wavelength	1550
*B* (Hz)	Reference bandwidth	30
*T* (K)	Temperature	293.15
*P*_0_ (μW)	Average optical power	5.85
*L* (km)	Length of fiber coil	2.8
*ν* (m/s)	Effective speed of light in the fiber	2.079 × 10^8^
$\phi_{0}$ (V)	Modulation phase	1.4
*A* (rad)	Modulation depth	1.8
*d* (μm)	Fiber cladding diameter	100
*κ* (W/(m K))	Thermal conductivity	1.02
*D_i_*(m^2^/s)	Thermal diffusity	0.82 × 10^−6^
*α_l_* (ppm/°C)	Linear thermal expansion coefficient	1.02
*dn_eff_/dT* (/°C)	Temperature coefficient of refractive index	9.9 × 10^−6^
*D* (cm)	Diameter of fiber coil	16
*n_eff_*	Effective refractive index	1.435
*W*_0_	Mode field radius	3.1
Δ*d* (μm)	Diameter of the air holes in the cladding	3.4
Δ*D* (μm)	Diameter of the two enlarged air holes in *x*-direction	5.8
Λ (μm)	Distance of two adjacent air hole centers	5.9
